# National Trends in Healthcare Expenditures for the Management of Skin Cancer in the United States

**DOI:** 10.1177/12034754241293131

**Published:** 2024-11-16

**Authors:** Bryan Ma, Matthew T. James, An-Wen Chan, P. Régine Mydlarski

**Affiliations:** 1 Division of Dermatology, Department of Medicine, Cumming School of Medicine, University of Calgary, Calgary, AB, Canada; 2 Division of Nephrology, Department of Medicine, Cumming School of Medicine, University of Calgary, Calgary, AB, Canada; 3 Women’s College Research Institute at Women’s College Hospital, Department of Medicine, University of Toronto, Toronto, ON, Canada

**Keywords:** melanoma, keratinocyte carcinoma, Global Burden of Disease (GBD) database, Institute for Health Metrics and Evaluations Disease Expenditure (DEX) 2016 project, health economics

## Abstract

**Background:**

Malignant melanoma and keratinocyte carcinomas account for a substantial proportion of healthcare expenditures in the United States.

**Objective:**

To estimate trends in the economic burden of skin cancer in the United States between 1996 and 2016.

**Methods:**

The Disease Expenditure Project and Global Burden of Disease databases were used to estimate annual total costs and population-standardized rates of change for skin cancer-related healthcare spending.

**Results:**

Skin cancer expenditures totaled $23.4 billion in 2016, of which $1.4 billion (95% CI: $1.3-$1.6 billion) was melanoma-related and $22 billion (95% CI: $18-$28 billion) was keratinocyte carcinoma-related. Most spending on skin cancer management occurred in ambulatory care settings [60.7% (95% CI: 57.7%-64.3%) for melanoma and 87.8% (95% CI: 87.2%-88.2%) for keratinocyte carcinoma]. Pharmaceutical costs for melanoma have increased since 2010 to $365 million (95% CI: $327-$416 million), which represents 26.1% (95% CI: 22.6%-29.3%) of total melanoma expenditure.

**Conclusions:**

Skin cancer management in the United States is costly. Expenditures have increased substantially since 1996 without signs of plateauing in recent years.

## Introduction

Skin cancer is the most common malignancy globally and its management represents a substantial public health burden.^1-3^ Despite preventative efforts, the incidence and prevalence of both melanoma and keratinocyte carcinoma continue to rise. From 1990 to 2019, the estimated incidence of melanoma and keratinocyte carcinoma in the United States increased from 12.6 and 402 to 17.0 per 100,000 persons/year and 787 per 100,000 persons/year, respectively. Furthermore, skin cancer is associated with considerable morbidity and mortality. The estimated age-standardized disability-adjusted life years (DALYs), defined as the years of full health lost due to disease-associated disability and premature mortality, in 2019 was 64.8 DALYs per 100,000 persons for melanoma and 26.8 DALYs per 100,000 persons for keratinocyte carcinoma.^1^

There has been little evaluation of healthcare expenditure on skin cancer in the United States over time. This is particularly important given major advancements in the diagnosis and management of dermatologic neoplasms over the past 20 years, including (a) the development of novel, targeted immunotherapies for melanoma, which have significantly improved outcomes in advanced disease^4-6^; (b) greater availability of effective precision surgical techniques such as Mohs micrographic surgery (MMS)^7,8^; and (c) shifts in healthcare infrastructure, provision, and insurance, altering how individuals present to and access medical care.^9,10^ Consequently, a better understanding of temporal trends in the costs and epidemiology of dermatologic neoplasms will better guide future resource allocation and public health policies.

We used nationally representative data to assess economic trends and underlying drivers of change in the cost of managing melanoma and keratinocyte carcinoma in the United States from 1996 to 2016.

## Materials and Methods

### Data Sources

Healthcare spending was estimated using publicly available data from the Institute for Health Metrics and Evaluations Disease Expenditure (DEX) 2016 project.^11^ The DEX project uses primary microdata from almost 6 billion unique public and private insurance claims, 150 million ambulatory or emergency department visits, and 6 million prescribed pharmaceuticals to approximate nearly 85% of United States healthcare spending annually between 1996 and 2016. Results are stratified into annual expenditure by age, sex, payer, and setting of care for over 150 medical conditions, including keratinocyte carcinoma and malignant melanoma. Estimates are adjusted for inflation and matched to official United States government figures reported by the National Health Expenditure Accounts. All values are listed in 2016 United States dollars. A complete summary of the DEX project and its methodology has been previously reported.^12-15^

Age and sex-stratified melanoma and keratinocyte carcinoma incidence data were obtained from the publicly available Institute for Health Metrics and Evaluations Global Burden of Disease (GBD) 2019 study.^16^ GBD is an international database with statistics on numerous epidemiologic measures (including incidence, prevalence, mortality, and DALYs) for 369 diseases and injuries across 204 countries. Annual data is available from 1990 to 2019. Primary microdata sources in the GBD include health surveys, peer-reviewed publication-reported statistics, and census tabulations with over 1700 citations and data sources for the United States alone.^17^

These 2 databases were used in combination to estimate the per capita cost (annual cost for each incident case) of malignant skin melanoma and keratinocyte carcinoma in the United States from 1996 to 2016. Both databases encode medical conditions using the *International Classification of Diseases 9th and 10th Revisions*, which have previously been shown to be accurate in identifying patients with skin cancer,^18^ allowing correlation of data between the 2 sources (Supplemental Table 1). Keratinocyte carcinoma includes squamous cell and basal cell carcinoma (BCC), the 2 most common forms of the disease.^1^

### Outcomes

The primary outcome was total annual expenditure on melanoma and keratinocyte carcinoma. This was stratified by age (<20, 20-44, 45-64, and ≥65 years), sex (male and female), payer (public insurance and private insurance), and setting of care (ambulatory, inpatient, nursing facility, pharmaceutical, and government administration). Ambulatory settings encompass all care provided in a physician’s office, freestanding clinic, or hospital outpatient department. Inpatient care includes all medications, diagnostic testing, and interventions utilized by inpatients for the duration of their hospitalization. Nursing facilities include nursing homes and other residential institutions, but not home-based care and hospices. Pharmaceutical expenditures are defined as all prescription medications purchased in a retail pharmacy setting and do not include medications provided over the counter or during emergency department or inpatient care. Administrative costs comprise all health protection, promotion, and disease prevention initiatives publicly funded by United States federal agencies.^14^

As a secondary outcome, we evaluated drivers of cost change over time, which were categorized as cost changes secondary to shifts in (a) total population, (b) population age, (c) disease incidence and prevalence, (d) service utilization, and (e) price and intensity of medical care. In ambulatory settings, service utilization is defined as mean visits per incident case, while service price and intensity are defined as mean spending per patient visit. Drivers of change data on DEX were available from 1996 to 2013.^12^

### Statistical Analysis

Estimates were produced annually over the full 20-year period. Incidence data were correlated with total annual expenditure to estimate cost per incident case. Trends in healthcare spending overtime were measured as population-standardized annualized rates of change, which is defined as the year-to-year percent change required to achieve the total spending change from 1996 to 2016 after adjusting for changes in population size, age, and sex structure. Population-standardized rates were calculated by multiplying the 2016 per-person spending rate for each age group and sex by the corresponding values in 1996 to approximate what spending would have been like in 1996 had the population had the characteristics of that of 2016.^13^ All analyses were performed using Stata 14.2 (StataCorp LLC, College Station, TX, USA).

## Results

### Total Costs

Supplemental Figures 1 and 2 summarize changes in total cost and incidence of melanoma and keratinocyte carcinoma from 1996 to 2016, respectively. Overall expenditures related to melanoma care rose from $773 million (95% confidence interval: $697-$890 million) in 1996 to $1.4 billion (95% CI: $1.3-$1.6 billion) in 2016, which represents a 3.0% annual increase in costs (95% CI: 2.3%-3.4%). Mean cost per incident case similarly increased from $15,361 in 1996 to $18,056 in 2016. Overall keratinocyte carcinoma expenditure in 1996 was $5.2 billion (95% CI: $4.1-$6.4 billion) compared to $22 billion (95% CI: $18-$28 billion) in 2016, a 7.3% annual increase (95% CI: 6.0%-9.0%). As with melanoma, cost per incident case increased from $3466 in 1996 to $5478 in 2016. Private insurance costs for skin cancer management represented the largest proportion of payment type [44.4% (95% CI: 36.4%-50.3%) vs 31.3% (95% CI: 26.2%-39.4%) public cost and 24.3% (95% CI: 18.9%-32.6%) out-of-pocket spending for melanoma, and 48.2% (95% CI: 42.6%-54.4%) private vs 45.1% (95% CI: 38.9%-50.9%) public and 6.6% (95% CI: 5.8%-7.9%) out-of-pocket spending for keratinocyte carcinoma].

### Costs by Age

The cost of melanoma and keratinocyte carcinoma were both highest in patients aged ≥65 years (Supplemental Table 2). Total spending in this age group in 2016 was 35.5% of all spending ($497 million, 95% CI: $423-$558 million) for melanoma, which represents a 2.1% (95% CI: 1.4%-2.6%) annual increase from $325 million (95% CI: $290-$372 million) since 1996. Those aged 45 to 64, 20 to 44, and <20 years accounted for $489 million (95% CI: $410-$565 million), $350 million (95% CI: $314-$417 million), and $61 million (95% CI: $53-$76 million) in expenditures, respectively.

The cost of managing keratinocyte carcinoma was also highest in populations aged ≥65 years, representing 59.1% of total annual spending in 2016 ($13 billion, 95% CI: $9.7-$16 billion). Costs in this age group have increased by 8.6% annually from $2.5 billion (95% CI: $1.8-$3.2 billion) in 1996 (95% CI: 6.8%-11.0%).

### Costs by Setting of Care

Most healthcare expenditure for skin cancer management occurs in an ambulatory setting [60.7% (95% CI: 57.7%-64.3%) for melanoma and 87.8% (95% CI: 87.2%-88.2%) for keratinocyte carcinoma], which has remained consistent over time (Supplemental Figures 3 and 4). However, prescribed pharmaceutical costs for melanoma increased from $2.1 million (95% CI: $1.2-$2.9 million) in 1996 to $365.0 million (95% CI: $327.0-$416.0 million) in 2016, a 29.6%/year increase. This represents the largest annual percentage increase in any healthcare expenditure category for the setting of care. Inpatient and nursing facility care collectively represent <5% of total costs and have remained stable over time.

### Costs by Sex

In 2016, healthcare expenditure for melanoma was higher in males compared to females [$793 million (95% CI: $697-$910 million) vs $608 million (95% CI: $523-$709 million)] (Supplemental Table 3). While the total cost of care for melanoma increased in both males and females from 1996 to 2016, the cost per incident case for males decreased from $17,812 to $16,775 over this same period, whereas the cost per incident case increased in females from $12,210 to $20,092.

For keratinocyte carcinoma, costs of management were higher in females than males. A total of $13 billion (95% CI: $9.9-$18 billion)in expenditures in 2016 were spent on females, which is an increase from $2.6 billion (95% CI: $1.9-$3.1 billion) in 1996. Similarly, the estimated cost per incident case in females rose from $3680 to $7928. In contrast, the cost of keratinocytes in males in 2016 was only $8.2 billion (95% CI: $6.3-$10.0 billion) compared to $2.6 billion (95% CI: $2.0-$3.4 billion) in 1996, although the cost per incident case remained stable ($3241 in 1996 to $3451 in 2016).

### Drivers of Change

Data on 5 drivers of changes were available between 1996 and 2013 (Supplemental Figure 5). The overall net cost of melanoma-related care rose by $460 million (5.3%), of which the majority was related to increases in the price and intensity of care provision, defined by mean cost per patient visit [$380 million/year (95% CI: $160-$600 million)]. Changes in population size, age, and disease incidence and prevalence also contributed to increasing costs, but service utilization fees (defined by mean visits per incident case) decreased by $600 million (95% CI: $95-$340 million) over 2 decades.

All drivers of change explored in this analysis increased the cost of managing keratinocyte carcinoma, for a net total change of $5.6 billion (22.5%). Unlike melanoma, the costs associated with changes in service utilization increased expenditure over time and were the single greatest factor in increasing total expenditures at $2.5 billion (95% CI $1.6-$3.5 billion).

## Discussion

In this study, we analyzed trends in healthcare expenditure for managing melanoma and keratinocyte carcinoma between 1996 and 2016 in the United States. The economic burden of both melanoma and keratinocyte carcinoma has risen significantly from $6.0 billion in 1996 to an estimated $23.4 billion in 2016. These findings are in keeping with other published estimates of the economic burden of skin cancer.^19,20^ We identified several important findings in this analysis. First, although the absolute spending for keratinocyte carcinoma is 16 times greater than melanoma, the cost per incident case of melanoma is 3 times greater, highlighting the complexities of caring for this population. Second, although most spending occurs in an ambulatory setting and in those aged ≥65 years, there has been a dramatic increase in pharmacologic spending for patients with melanoma, coinciding with the development of immune checkpoint inhibitors that have revolutionized melanoma care. Finally, different factors are contributing to changes in spending for skin cancer management, whereas patients with keratinocyte carcinoma are increasing their service utilization, it is the increasing price of care that is the greatest contributing factor in patients with melanoma.

Pharmaceutical care costs have increased significantly for melanoma, primarily after 2010. This coincides with the development of targeted immunotherapies for metastatic melanoma, which have transformed the management of advanced-stage disease. Ipilimumab received Food and Drug Agency (FDA) approval for the management of advanced melanoma in 2011, whereas both nivolumab and pembrolizumab received approval in 2014. While these therapies improve both progression-free and overall survival,^4,6,21-23^ these medications are also extremely expensive. The cost of nivolumab-ipilimumab combination therapy in patients with non-small cell lung cancer has been estimated at $377,400 per year.^24,25^ The widespread adoption of immunotherapy likely represents one of the primary causes of increasing costs secondary to rising prices per incident case of melanoma. Interestingly, vismodegib received FDA approval in 2012 for the treatment of recurrent, locoregionally advanced, or metastatic BCC. However, our data do not show a similar rise in pharmaceutical costs for managing BCC as seen with the approval of immune checkpoint inhibitors for melanoma. This may be due to the relatively lower prevalence of advanced BCC, or the slower uptake of the drug compared to the more widely adopted treatments for melanoma.

Therefore, further development of potentially cost-saving medication measures (such as biosimilar agents) may be important in controlling future pharmaceutical expenditures. However, the total economic impact of immunotherapies is challenging to estimate. These therapies are associated with an increased risk of immune-mediated adverse events, such as bullous pemphigoid with anti-PD-1 agents, which may require potentially expensive inpatient care.^26^ While the widespread uptake of PD-1 inhibitors after 2014 is not captured in our drivers of cost change analysis, we would postulate that these agents likely further increased service utilization and intensity of care-related costs in melanoma due to their associated need for surveillance, diagnosis, and management of cutaneous and systemic immune-mediated adverse events.

Higher costs in ambulatory settings are likely related to advancements in outpatient keratinocyte carcinoma management. Therapeutic options for keratinocyte carcinoma, including surgical excision, curettage and electrodesiccation, photodynamic therapy, cryotherapy, and topical 5-fluorouracil, are all performed in ambulatory settings.^27,28^ In-office MMS is also a mainstay of treatment^29^: it is estimated that the rate of MMS increased by approximately 20% in Medicare beneficiaries from 2012 to 2017, and this was a primary driver in the nearly 10% increase in skin cancer procedure-related expenditures over the same time period.^30^

Skin cancer pathogenesis is complex and multifactorial. Progressive accumulation of modifiable environmental risk factors, such as ultraviolet light exposure, plays a crucial role.^31^ This is likely an underlying driver of increased incidence and related cost of skin cancer in elderly patients who have been exposed to the sun for longer periods by virtue of their age. Given the increasing incidence and prevalence of melanoma and keratinocyte carcinoma in the United States,^1^ population-wide preventative measures targeted at younger populations are essential in minimizing the incidence of disease and facilitating diagnosis during early stages.^32^ The American Academy of Dermatology’s SPOTme skin cancer campaign is one of the largest active public screening programs for malignancy. Over a 30-year period, more than 2 million Americans have been screened and an estimated 180,000 melanoma or keratinocyte carcinomas have been detected. Despite the success of these campaigns,^33^ Aggarwal et al demonstrated that the incidence of skin cancer continues to increase in the United States, suggesting that more aggressive population-level screening and education campaigns are still required.^1^

This study uses nationally representative data to estimate cost and economic temporal trends across numerous demographic groups and settings of care for skin cancer over a 20-year period. However, this study also has several limitations. First, skin cancer data is presented in aggregate with no specificity based on tumor stage. This limits analysis of incidence and cost-effectiveness of lower-level therapies appropriate for more localized disease versus systemic therapies indicated in advanced malignancy, and hampers identification of which patient populations require intensive and prolonged management. Similarly, patients with advanced disease likely represent a disproportionate share of the reported costs as this population will require much more intensive diagnostic investigations and therapeutic interventions. Second, data from DEX and GBD databases are limited by estimates associated with mathematical modeling and ICD code-based studies, and we assume that the cases captured in the GBD are the same cases as represented in the DEX, which cannot be verified. Moreover, we are unable to identify potential causes for specific observed trends. For instance, our results suggest the incidence of keratinocyte carcinomas increased in the early 2000s. Other published studies have reported similar findings in specific populations and geographies during that same period.^34^ However, it is difficult to ascertain whether these findings result from increased skin cancer detection rates, changes in risk factor exposures, or statistical modeling. Finally, data from the DEX project only estimates direct costs and does not include the indirect costs (defined as loss of resources and opportunities due to disease) associated with skin cancer, such as disease and pharmacotherapy-related morbidity. Therefore, we likely underestimate the true cost of skin cancer.

In conclusion, the management of skin cancer represents a substantial economic burden on the healthcare system in the United States. The etiologic drivers of rising costs differ between melanoma and keratinocyte carcinoma, wherein the former is mostly secondary to increasing price and intensity of therapy while the latter is due to expanding service utilization. These may be reflections of advancements in immunotherapies for melanoma and increasing incidence of multiple, recurrent keratinocyte carcinomas, respectively. These data may better inform ongoing public health and preventative efforts to control the cost of managing skin cancer while optimizing patient outcomes.

## Supplemental Material

sj-docx-1-cms-10.1177_12034754241293131 – Supplemental material for National Trends in Healthcare Expenditures for the Management of Skin Cancer in the United States Supplemental material, sj-docx-1-cms-10.1177_12034754241293131 for National Trends in Healthcare Expenditures for the Management of Skin Cancer in the United States by Bryan Ma, Matthew T. James, An-Wen Chan and P. Régine Mydlarski in Journal of Cutaneous Medicine and Surgery

sj-png-2-cms-10.1177_12034754241293131 – Supplemental material for National Trends in Healthcare Expenditures for the Management of Skin Cancer in the United States Supplemental material, sj-png-2-cms-10.1177_12034754241293131 for National Trends in Healthcare Expenditures for the Management of Skin Cancer in the United States by Bryan Ma, Matthew T. James, An-Wen Chan and P. Régine Mydlarski in Journal of Cutaneous Medicine and Surgery

sj-png-3-cms-10.1177_12034754241293131 – Supplemental material for National Trends in Healthcare Expenditures for the Management of Skin Cancer in the United States Supplemental material, sj-png-3-cms-10.1177_12034754241293131 for National Trends in Healthcare Expenditures for the Management of Skin Cancer in the United States by Bryan Ma, Matthew T. James, An-Wen Chan and P. Régine Mydlarski in Journal of Cutaneous Medicine and Surgery

sj-png-4-cms-10.1177_12034754241293131 – Supplemental material for National Trends in Healthcare Expenditures for the Management of Skin Cancer in the United States Supplemental material, sj-png-4-cms-10.1177_12034754241293131 for National Trends in Healthcare Expenditures for the Management of Skin Cancer in the United States by Bryan Ma, Matthew T. James, An-Wen Chan and P. Régine Mydlarski in Journal of Cutaneous Medicine and Surgery

sj-png-5-cms-10.1177_12034754241293131 – Supplemental material for National Trends in Healthcare Expenditures for the Management of Skin Cancer in the United States Supplemental material, sj-png-5-cms-10.1177_12034754241293131 for National Trends in Healthcare Expenditures for the Management of Skin Cancer in the United States by Bryan Ma, Matthew T. James, An-Wen Chan and P. Régine Mydlarski in Journal of Cutaneous Medicine and Surgery

sj-png-6-cms-10.1177_12034754241293131 – Supplemental material for National Trends in Healthcare Expenditures for the Management of Skin Cancer in the United States Supplemental material, sj-png-6-cms-10.1177_12034754241293131 for National Trends in Healthcare Expenditures for the Management of Skin Cancer in the United States by Bryan Ma, Matthew T. James, An-Wen Chan and P. Régine Mydlarski in Journal of Cutaneous Medicine and Surgery
