# Supplementary material for: National Trends in Healthcare Expenditures for the Management of Skin Cancer in the United States
Source: J Cutan Med Surg. 2024 Nov 16;29(1):33–8. doi: 10.1177/12034754241293131 (PMC11829503; doi:10.1177/12034754241293131)
Supplement: sj-docx-1-cms-10.1177_12034754241293131 – Supplemental material for National Trends in Healthcare Expenditures for the Management of Skin Cancer in the United States [file sj-docx-1-cms-10.1177_12034754241293131.docx]

**Supplementary Table 1**: ICD codes for malignant skin melanoma and keratinocyte carcinoma used by the Disease Expenditure and Global Burden of Disease databases.

| Cause | ICD-9 Code(s) | ICD-10 Code(s) |
| --- | --- | --- |
| Melanoma | 172, 172.0, 172.1, 172.2, 172.3, 172.4,  172.5, 172.6, 172.7, 172.8, 172. | C43, C43.0, C43.1, C43.10, C43.11, C43.12, C43.2, C43.20, C43.21,  C43.22, C43.3, C43.30, C43.31, C43.39, C43.4, C43.5, C43.51,  C43.52, C43.59, C43.6, C43.60, C43.61, C43.62, C43.7, C43.70, C43.71, C43.72, C43.8, C43.9, D03.0, D03.4, D03.8, D03.9 |
| Keratinocyte carcinoma | 173, 173.0, 173.00, 173.01, 173.02,  173.09, 173.1, 173.10, 173.11, 173.12,  173.19, 173.2, 173.20, 173.21, 173.22,  173.29, 173.3, 173.30, 173.31, 173.32,  173.39, 173.4, 173.40, 173.41, 173.42,  173.49, 173.5, 173.50, 173.51, 173.52,  173.59, 173.6, 173.60, 173.61, 173.62,  173.69, 173.7, 173.70, 173.71, 173.72,  173.79, 173.8, 173.80, 173.81, 173.82,  173.89, 173.9, 173.90, 173.91, 173.92,  173.99, 209.31, 209.32, 209.33,  209.34, 209.35, 209.36, 214, 214.0,  214.1, 215, 215.0, 215.2, 215.3, 215.4,  215.5, 215.6, 215.7, 215.8, 215.9, 216,  216.0, 216.1, 216.2, 216.3, 216.4,  216.5, 216.6, 216.7, 216.8, 216.9,  222.4, 232, 232.0, 232.1, 232.2, 232.3,  232.4, 232.5, 232.6, 232.7, 232.8,  232.9, 238.2, V76.43 | C44.1, C44.10, C44.11, C44.111, C44.112, C44.119, C44.12, C44.121,  C44.122, C44.129, C44.19, C44.2, C44.20, C44.21, C44.211, C44.212,  C44.219, C44.22, C44.221, C44.222, C44.229, C44.29, C44.3, C44.30,  C44.31, C44.310, C44.311, C44.319, C44.32, C44.320, C44.321, C44.329,  C44.39, C44.4, C44.41, C44.42, C44.5, C44.50, C44.51, C44.510,  C44.511, C44.519, C44.52, C44.520, C44.521, C44.529, C44.59, C44.6,  C44.60, C44.601, C44.602, C44.609, C44.61, C44.611, C44.612, C44.619,  C44.62, C44.621, C44.622, C44.629, C44.69, C44.7, C44.70, C44.71,  C44.711, C44.712, C44.719, C44.72, C44.721, C44.722, C44.729, C44.79,  C44.8, C44.81, C44.82, C44.9, C44.91, C44.92, D04.0, D04.4, D04.5,  D04.8, D04.9, D17.0, D17.1, D21.0, D21.3, D21.4, D21.5, D21.6, D21.9,  D22.0, D22.4, D22.5, D22.9, D23.0, D23.4, D23.5, D23.9, D29.4, D48.5,  C44.101, C44.102, C44.109, C44.191, C44.192, C44.199, C44.201, C44.202, C44.209, C44.291, C44.292, C44.299, C44.300, C44.301, C44.309, C44.390, C44.391, C44.399, C44.40, C44.49, C44.500, C44.501, C44.509, C44.590, C44.591, C44.599, C44.691, C44.692, C44.699, C44.701, C44.702, C44.709, C44.791, C44.792, C44.799, C44.80,  C44.89, C44.90, C44.99 |

ICD, international classification of disease

| Supplementary Table 2: Total healthcare expenditure on skin cancer in 2016 stratified by age | | | | | | | | | | | | | |
| --- | --- | --- | --- | --- | --- | --- | --- | --- | --- | --- | --- | --- | --- |
|  | **Age (years)** | **Ambulatory** | | **Inpatient** | | **Prescribed pharmaceutical** | | **Government administration** | | **Nursing facility care** | | **All settings of care** | |
|  |  | 2016 (USD, millions) (95% CI) | Annual % change from 1996 | 2016 (USD, millions, 95% CI) | Annual % change from 1996 | 2016 (USD, millions, 95% CI) | Annual % change from 1996 | 2016 (USD, millions, 95% CI) | Annual % change from 1996 | 2016 (USD, millions, 95% CI) | Annual % change from 1996 | 2016 (USD, millions, 95% CI) | Annual % change from 1996 |
| Melanoma | <20 | 27 (21 – 41) | 0.25 | 0.55 (0.27 – 1.3) | 0.59 | 30 (26 – 35) | 30.42 | 3.9 (2.3 – 5.6) | 2.75 | 0.01 (0.00 – 0.15) | 9.49 | 61 (53 – 76) | 3.92 |
|  | 20 – 44 | 172 (145 – 224) | 0.39 | 7.6 (5.2 – 11) | -4.32 | 150 (132 – 174) | 30.58 | 25 (18 – 32) | 1.54 | 0.07 (0.04 – 0.10) | 2.24 | 350 (314 – 417) | 3.00 |
|  | 45 – 64 | 303 (229 – 361) | 2.93 | 24 (19 – 29) | -0.93 | 120 (104 – 139) | 30.63 | 42 (29 – 52) | 3.20 | 0.66 (0.45 – 0.93) | 5.83 | 489 (410 – 565) | 4.01 |
|  | ≥65 | 349 (284 – 406) | 1.15 | 25 (21 – 30) | -0.16 | 66 (61 – 72) | 26.28 | 46 (38 – 53) | 5.45 | 10 (7.9 – 15.0) | 3.79 | 497 (423 – 558) | 2.14 |
| Keratinocyte Carcinoma | <20 | 89 (20 – 217) | -0.38 | 1.8 (0.89 – 2.60) | -1.50 | 10 (8.4 – 12) | 7.69 | 11 (3.4 – 26.0) | 0.21 | 0.19 (0.10 – 0.39) | -2.49 | 113 (35 – 254) | 0.026 |
|  | 20 – 44 | 1,100 (755 – 1,500) | 0.66 | 26 (20 – 32) | -1.40 | 51 (42 – 58) | 3.32 | 139 (96 – 181) | 0.99 | 0.21 (0.09 – 0.38) | -0.48 | 1,340 (937 – 1,700) | 0.73 |
|  | 45 – 64 | 6,400 (4,900 – 8,400) | 8.48 | 107 (87 – 130) | 2.80 | 47 (35 – 71) | 3.81 | 756 (565 – 983) | 8.65 | 2.0 (1.6 – 2.5) | 6.69 | 7,300 (5,600 – 9,500) | 8.30 |
|  | ≥65 | 11,000 (8,600 – 14,000) | 8.53 | 118 (99 – 149) | 1.47 | 59 (36 – 89) | 4.49 | 1,300 (964 – 1,600) | 11.63 | 28 (22 – 41) | 2.56 | 13,000 (9,700 – 16,000) | 8.56 |

CI, confidence interval; USD, United States Dollars

| Supplementary Table 3: Total healthcare expenditure on melanoma in 2016 stratified by sex | | | | | | | | | | | | | |
| --- | --- | --- | --- | --- | --- | --- | --- | --- | --- | --- | --- | --- | --- |
|  | **Sex** | **Ambulatory** | | **Inpatient** | | **Prescribed pharmaceutical** | | **Government administration** | | **Nursing facility care** | | **All settings of care** | |
|  |  | 2016 (USD, millions) (95% CI) | Annual % change from 1996 | 2016 (USD, millions, 95% CI) | Annual % change from 1996 | 2016 (USD, millions, 95% CI) | Annual % change from 1996 | 2016 (USD, millions, 95% CI) | Annual % change from 1996 | 2016 (USD, millions, 95% CI) | Annual % change from 1996 | 2016 (USD, millions, 95% CI) | Annual % change from 1996 |
| Melanoma | Men | 506 (422 – 610) | 0.96 | 36 (30 – 42) | -0.78 | 178 (159 – 203) | 27.57 | 67 (53 – 82) | 2.92 | 6.3 (4.8 – 8.9) | 5.48 | 793 (697 – 910) | 2.32 |
|  | Women | 346 (274 – 431) | 2.41 | 22 (17 – 26) | -1.92 | 187 (170 – 212) | 32.31 | 49 (38 – 59) | 4.41 | 4.7 (3.6 – 6.5) | 2.33 | 608 (523 – 709) | 4.12 |
| Keratinocyte Carcinoma | Men | 7,100 (5,400 – 9,140) | 5.83 | 166 (141 – 200) | 2.45 | 81 (63 – 92) | 4.22 | 825 (646 – 1,100) | 7.50 | 17 (13 – 24) | 3.77 | 8,200 (6,300 – 10,000) | 5.85 |
|  | Women | 12,000 (8,700 – 15,000) | 8.65 | 85 (72 – 102) | 0.14 | 86 (59 – 131) | 3.89 | 1,400 (1,000 – 1,800) | 9.62 | 14 (10 – 19) | 1.67 | 13,000 (9,900 – 18,000) | 8.53 |

CI, confidence interval; USD, United States Dollars

| Supplementary Table 4: Total healthcare expenditure on skin cancer in 2016 stratified by payer | | | | | | | | | | | | | |
| --- | --- | --- | --- | --- | --- | --- | --- | --- | --- | --- | --- | --- | --- |
|  | **Insurance** | **Ambulatory** | | **Inpatient** | | **Prescribed pharmaceutical** | | **Government administration** | | **Nursing facility care** | | **All settings of care** | |
|  |  | 2016 (USD, millions) (95% CI) | Annual % change from 1996 | 2016 (USD, millions, 95% CI) | Annual % change from 1996 | 2016 (USD, millions, 95% CI) | Annual % change from 1996 | 2016 (USD, millions, 95% CI) | Annual % change from 1996 | 2016 (USD, millions, 95% CI) | Annual % change from 1996 | 2016 (USD, millions, 95% CI) | Annual % change from 1996 |
| Melanoma | Public | 301 (239 – 390) | 0.83 | 25 (21 – 33) | -0.03 | 63 (25 – 130) | 33.80 | 43 (35 – 57) | 6.61 | 6.5 (4.3 – 8.2) | 1.52 | 438 (351 – 576) | 1.99 |
|  | Private | 464 (338 – 565) | 1.97 | 31 (22 – 38) | -1.97 | 53 (9.46 – 136.0) | 25.92 | 73 (53 – 90) | 2.28 | 3 (0.72 – 7.20) | 5.96 | 624 (456 – 772) | 2.14 |
| Keratinocyte Carcinoma | Public | 8,600 (6,600 – 11,000) | 8.59 | 96 (52 – 124) | 1.25 | 35 (22 – 54) | 10.06 | 960 (730 – 1,300) | 13.66 | 18 (12 – 23) | 2.34 | 9,700 (7,500 – 13,000) | 8.73 |
|  | Private | 9,000 (7,100 – 11,000) | 6.62 | 146 (106 – 206) | 1.95 | 70 (33 – 103) | 6.34 | 1,200 (972 – 1,500) | 6.66 | 8.2 (1.8 – 20) | 4.73 | 10,000 (8,310 – 13,000) | 6.51 |

CI, confidence interval; USD, United States Dollars
