# Supplementary figures and images for: National Trends in Healthcare Expenditures for the Management of Skin Cancer in the United States
Source: J Cutan Med Surg. 2024 Nov 16;29(1):33–8. doi: 10.1177/12034754241293131 (PMC11829503; doi:10.1177/12034754241293131)

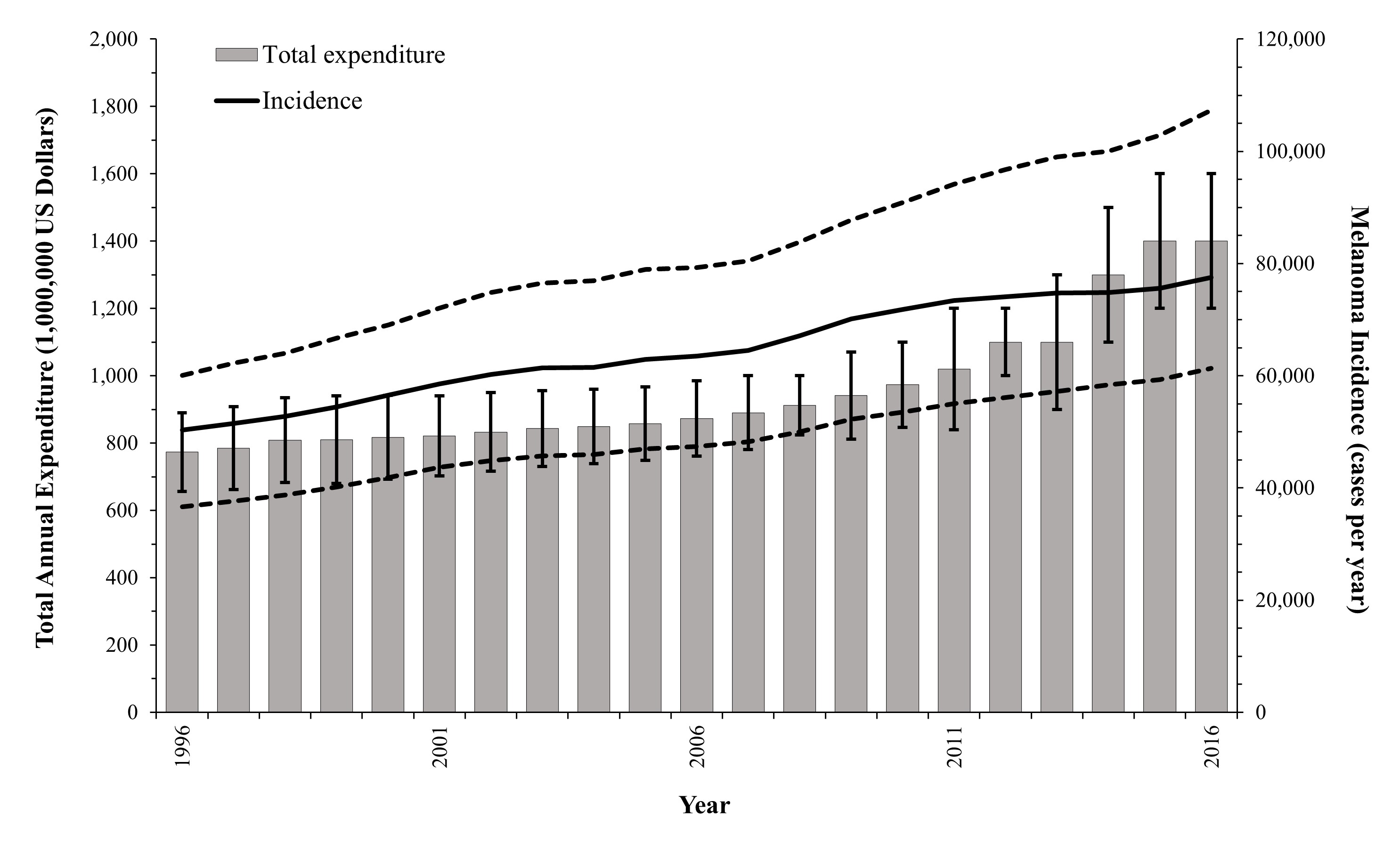

Supplement: sj-png-2-cms-10.1177_12034754241293131 – Supplemental material for National Trends in Healthcare Expenditures for the Management of Skin Cancer in the United States [file sj-png-2-cms-10.1177_12034754241293131.jpg]

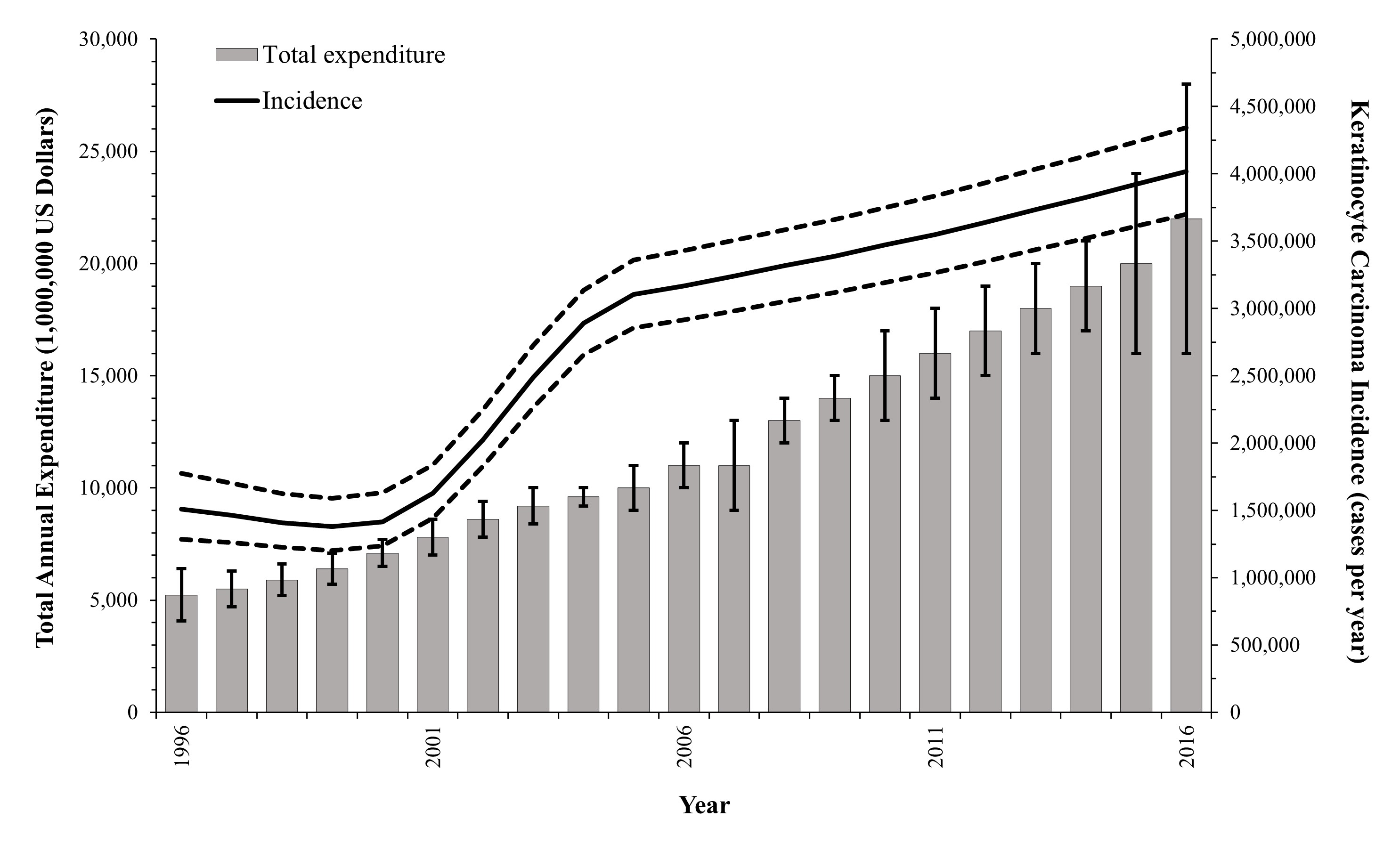

Supplement: sj-png-3-cms-10.1177_12034754241293131 – Supplemental material for National Trends in Healthcare Expenditures for the Management of Skin Cancer in the United States [file sj-png-3-cms-10.1177_12034754241293131.jpg]

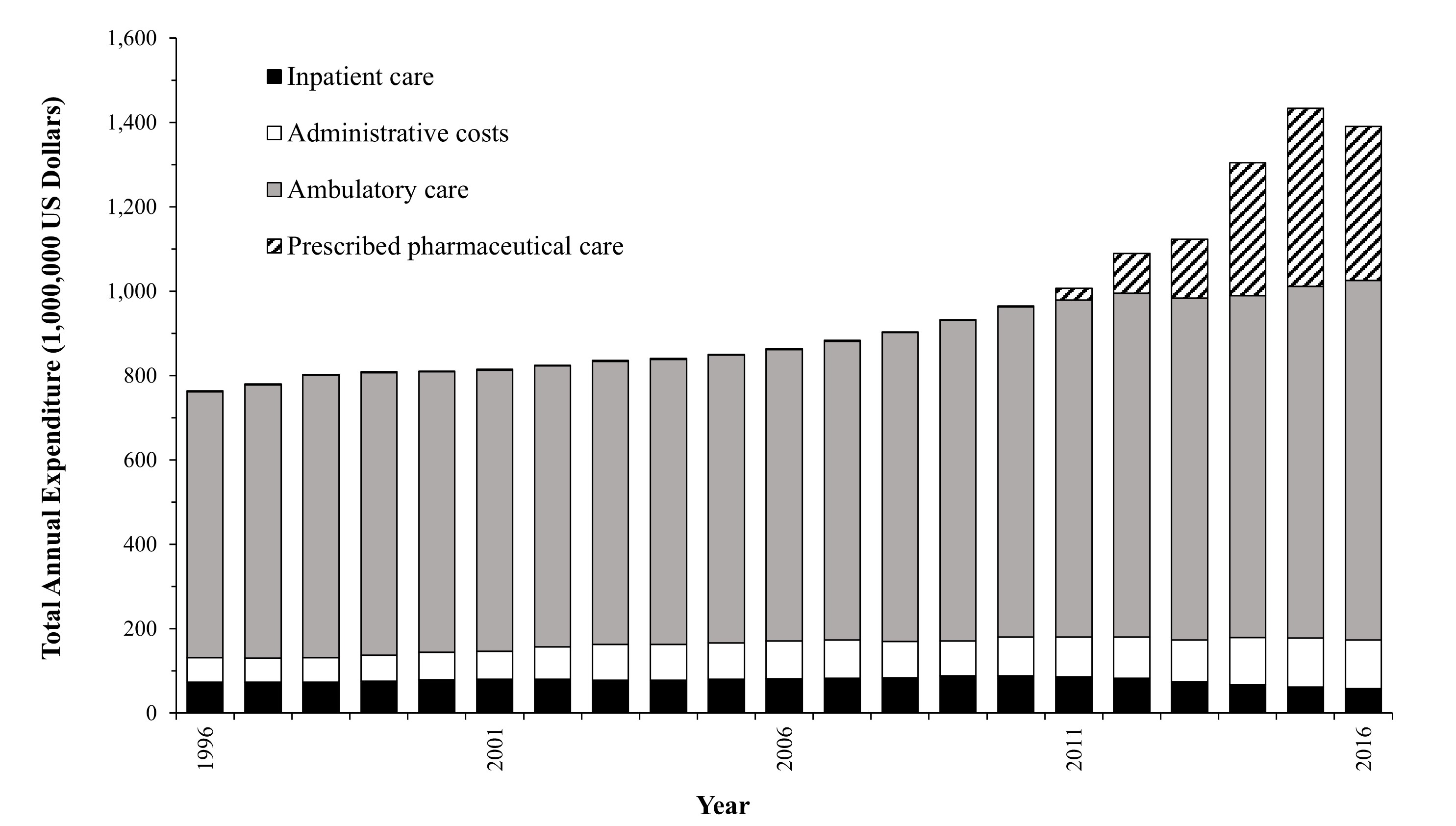

Supplement: sj-png-4-cms-10.1177_12034754241293131 – Supplemental material for National Trends in Healthcare Expenditures for the Management of Skin Cancer in the United States [file sj-png-4-cms-10.1177_12034754241293131.jpg]

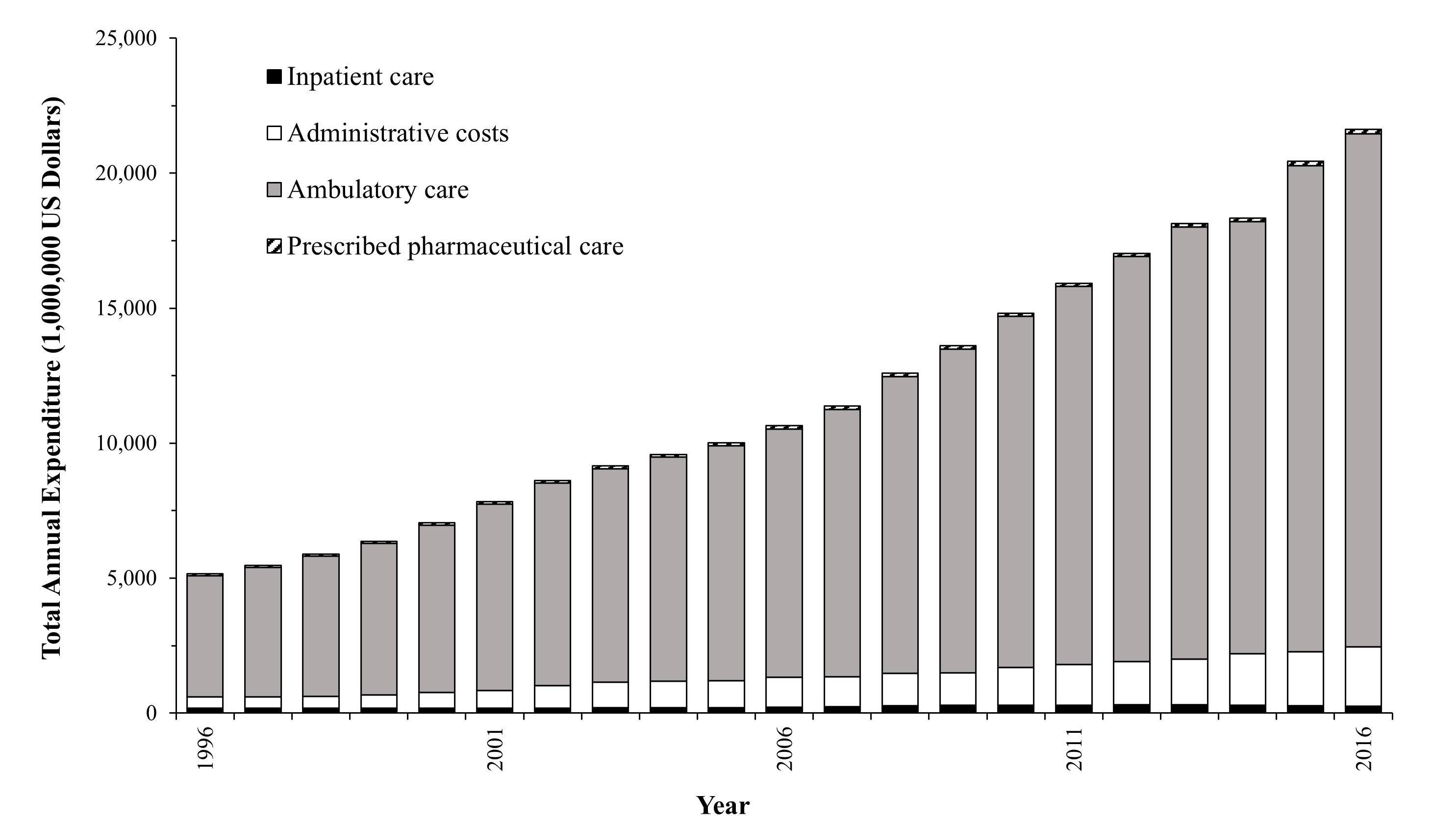

Supplement: sj-png-5-cms-10.1177_12034754241293131 – Supplemental material for National Trends in Healthcare Expenditures for the Management of Skin Cancer in the United States [file sj-png-5-cms-10.1177_12034754241293131.jpg]

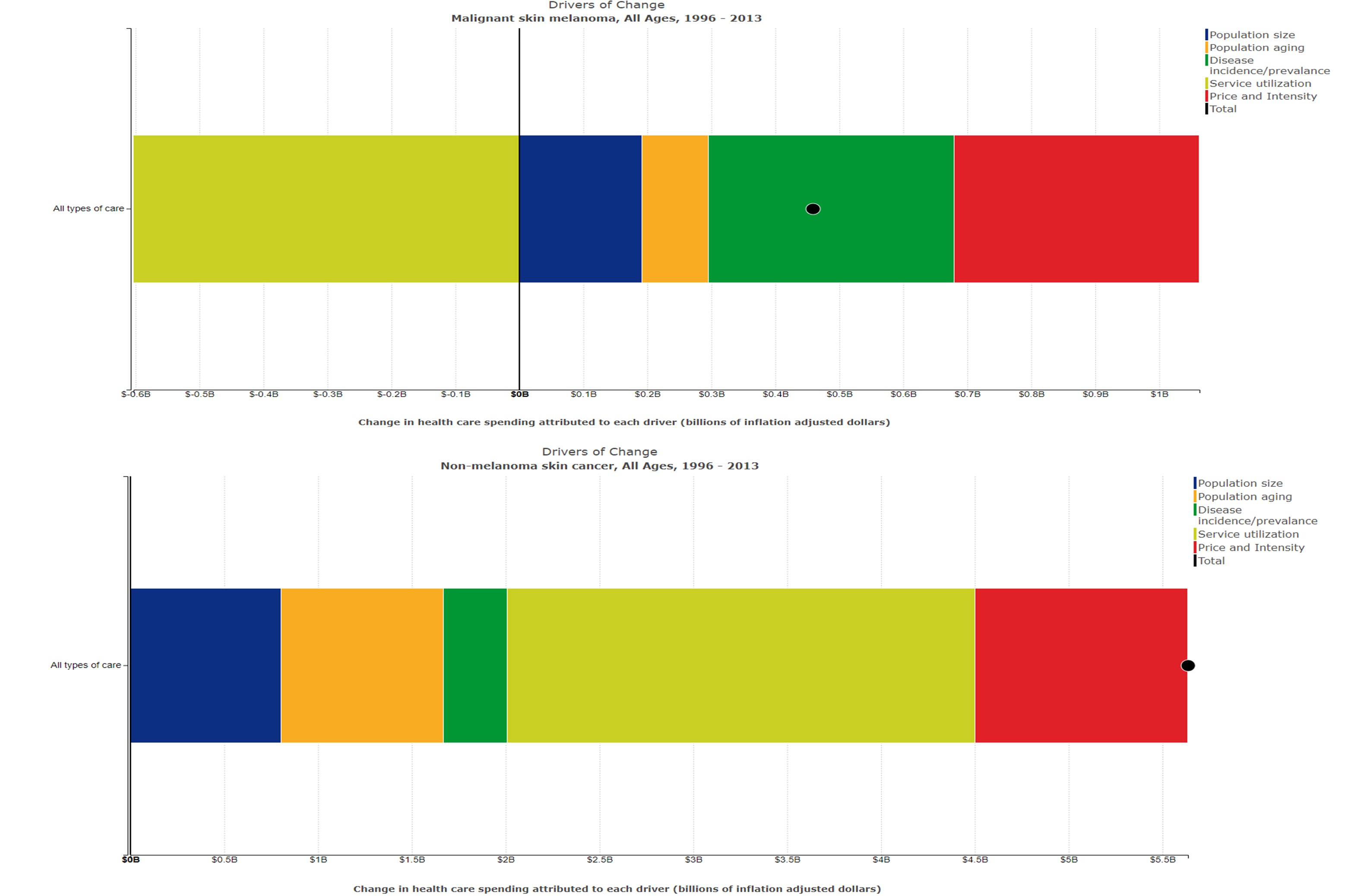

Supplement: sj-png-6-cms-10.1177_12034754241293131 – Supplemental material for National Trends in Healthcare Expenditures for the Management of Skin Cancer in the United States [file sj-png-6-cms-10.1177_12034754241293131.jpg]
